# Optimized Quantitative Bacterial Two-Hybrid (qB2H) for Protein–Protein Interaction Assessment

**DOI:** 10.34133/csbj.0098

**Published:** 2026-05-25

**Authors:** Antoine Guyot, Emma Maillard, Kelly Ferreira-Pinto, Laure Plançon-Arnould, Aravindan Arun Nadaradjane, Raphaël Guérois, Francoise Ochsenbein, Loïc Martin, Oscar Henrique Pereira Ramos

**Affiliations:** ^1^ Sanofi Large Molecule Research, Vitry-sur-Seine, France.; ^2^ CEA, Département Médicaments et Technologies pour la Santé (DMTS), SIMoS, Gif-sur-Yvette, France.; ^3^ Université Paris-Saclay, 91191 Gif-sur-Yvette, France.; ^4^ Université Paris-Saclay, CEA, CNRS, Institute for Integrative Biology of the Cell (I2BC), 91198 Gif-sur-Yvette, France.

## Abstract

Characterizing mutation effects on protein–protein interactions (PPIs) is crucial for elucidating protein structure and function. Massively parallel PPI variant analyses such as deep mutational scanning enable interface identification and generate datasets for machine learning. *In cellulo* strategies such as two-hybrid systems provide straightforward access to such data, but reliability depends on quantitative properties. Here, we show that existing bacterial two-hybrid (B2H) systems have limitations constraining accurate dataset generation. We engineered and benchmarked optimized quantitative B2H (qB2H) alternatives, enabling strain-independent assays, improved metrics, and generation of high-quality datasets. We demonstrate qB2H utility through interface mapping and binder optimization. Perturbation analysis of single-site variants accurately recovered known antisilencing function 1 (ASF1) complex contact positions, matching crystallographic data. Integration of generative artificial-intelligence-based design yielded an ASF1-binding peptide with a 70-fold increase in affinity. qB2H offers to R&D scientists a robust, reusable platform for quantitative PPI analysis, enabling both rational protein engineering and data-driven discovery. Code, data, and materials were made available to the community.

## Graphical Abstract

**Figure:**
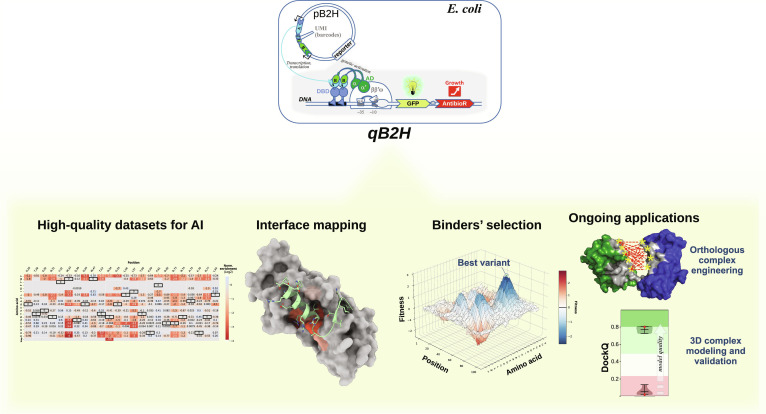

## Introduction

Protein–protein interactions (PPIs) are fundamental to biological processes, underpinning fields from biochemistry and physiology to drug discovery. Analyzing protein complexes through mutation effects yields invaluable insights by pinpointing interface positions, quantifying residue contributions to binding strength, and identifying hotspots. Incorporated into machine learning models, these mutational effect datasets guide molecular optimization [1–3] across research, diagnostics, and therapeutic development with potential broad societal benefits. These applications rely on precise and reliable readouts. Beyond single point mutants, the possibility of simultaneously mutating 2 complex partners can provide important clues about compensatory effects and the basis of binding specificity. However, few robust, quantitative, and user-friendly technologies exist to screen for high-throughput simultaneous variations in 2 partners within the same assay [4] (dual-partner deep mutational scanning [dpDMS]). While *in vitro* approaches allow investigating complex formation under arbitrary and well-defined conditions, they can be burdensome for achieving higher throughput. *In cellulo* approaches [5] are often cost-effective, compatible with high-throughput screening and can be coupled with next-generation sequencing (NGS) for massive data analysis. Protein complementation assays (PCAs) and two-hybrid (2H) systems are among the approaches suitable for large-scale dpDMS screening [6,7]. While PCAs provide simpler and more direct readouts, 2H systems offer greater flexibility in selecting the nature of the generated signal.

The concept of 2H was first introduced in yeast [8], followed by bacteria [9] (B2H) and mammalian cells [10]. While yeast 2H and mammalian cell 2H support posttranslational modifications, B2H is considered more resilient to false results [9,11] for eukaryotic complexes and better fitted for fast screening of large libraries. Furthermore, the B2H system can be used even for multimeric proteins [7,12], secreted proteins [11], proteins with posttranslational modifications (if the latter are not required for the interaction) [11], and bacterial cells are convenient for synthetic systems engineering [13–15]. Some B2H systems rely on the coexpression of 2 fusion proteins: the bait, which includes a DNA binding domain (e.g., the λ cI [a λ bacteriophage DNA binding protein] repressor fused to one interaction partner), and the prey, which carries a transcriptional activation domain (e.g., the α [*rpoA*] or ω [*rpoZ*] subunit of bacterial RNA polymerase fused to the other partner). Interaction between these hybrid proteins recruits RNA polymerase to a synthetic promoter, thereby inducing the transcription of one or more reporter genes. Hochschild and colleagues [9] pioneered such B2H setup in 1997. The entire system included 2 plasmids (pBRα-*rpoA* and pAC-*cI*) and a strain holding a B2H-responsive promoter that controlled *lacZ* gene expression. Later, Ranganathan and colleagues [16] proposed an alternative system implemented in 3 plasmids (pZA31-RNAα, pZS22, and pZE1RM) and used enhanced green fluorescent protein (eGFP) as reporter. Based on the characteristics of these B2H systems, we reasoned that Hochschild’s system would provide low signal output, while Ranganathan’s setup could impose a metabolic burden due to the number of plasmids (and antibiotics for plasmid selection).

Here, we report the engineering, characterization, and application of a convenient and robust quantitative B2H (qB2H) system designed to serve as a versatile tool for future protein engineering projects. First, we describe the engineering strategy by evaluating alternative versions of the pivotal B2H-responsive promoter—which responds to complex formation (cI–partner_A and rpoA–partner_B)—within a harmonized reporter context, to characterize their properties and identify options for improving B2H robustness and quantitativeness. Next, we validate the optimized systems in representative applications, including interface mapping and binder optimization. As a model system for qB2H engineering, benchmarking, and validation, we used complexes between the histone chaperone ASF1 (antisilencing function 1) and synthetic peptides whose dissociation constants (*K*_d_) had been previously determined by isothermal titration calorimetry (ITC), covering a range from undetectable binding (>100 μM) to nanomolar affinities [17]. These peptides have been originally designed as first steps toward the development of anticancer compounds impairing the proliferation of cancer cell lines [17–19]. Senior authors of these publications are also involved in the present work, guaranteeing consistency in the *K*_d_ measurement methodology and its associated uncertainties as applied here.

## Results

### qB2H engineering provides improved *K*_d_ *vs.* signal profile and quantitative metrics for binding affinity assessment

An optimized qB2H system can advance protein engineering and expand its integration with machine learning applications. To achieve this, it is essential to identify current limitations and enhance qB2H signal quantification in relation to the thermodynamic properties of specific protein interactions. We addressed key factors limiting signal quantification—plasmid architecture, expression balance, methylation, and reporter design—resulting in 4 qB2H versions (v1 to v4; Table 1).

**Table 1.** Summary of engineered B2H features across versions (v1 to v4; details on Fig. S1A [plasmid genealogy] and Fig. S1B [plasmid maps])

Feature	qB2H versions			
	v1	v2	v3	v4
Number of plasmids	2	1	1	1
*cI* fusion promoter	lac_UV5	lac_UV5	L_tetO	L_tetO
*cI* RBS (T.I.R.) ^a^	3,625	3,625	371	51
qB2H promoter	OL2-62_L ^b^	OL2-62_L	OL2-62_L_∆Dcm	OL2-62_L_∆Dcm
Reporter	Strong RBS-***eGFP***-strong RBS-***kanR***	Strong RBS-***eGFP***-strong RBS-***kanR***	Weak RBS-***eGFP***-weak RBS-***kanR***	Weak RBS-***kanR***

^**a**^ T.I.R.s were estimated using “Promoter Calculator” from “De Novo DNA” server (www.denovodna.com).

^b^ Alternative promoters were evaluated in the context of v1 series plasmids, but OL2-62_L was assumed as reference.

Bolded terms were used to highlight protein coding elements.

**Fig. 1.**
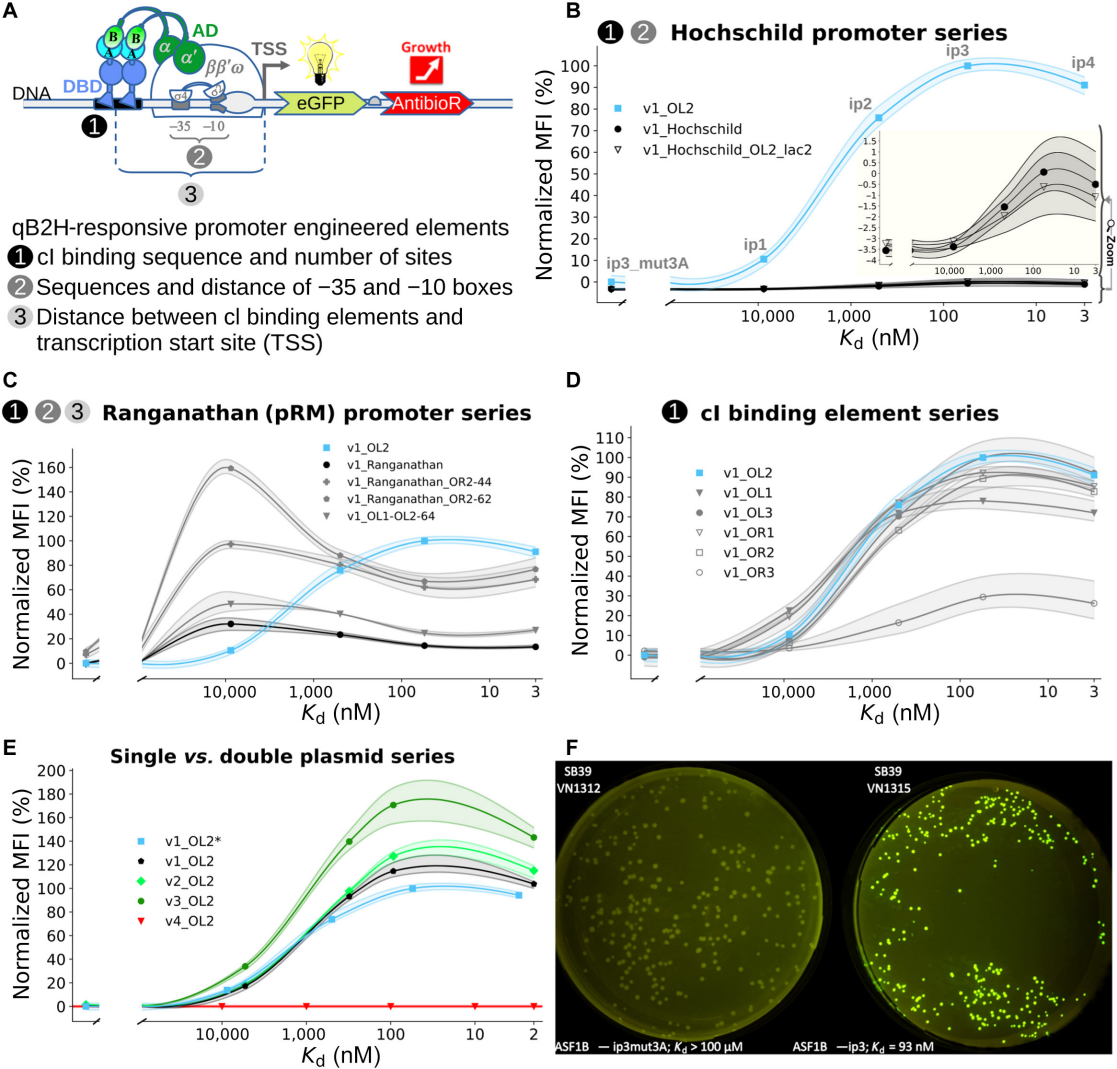
Bacterial two-hybrid (B2H) engineering. Details on B2H-responsive promoters are provided in Table S2. (A) Schematic of the quantitative B2H (qB2H) reporter gene showing promoter structure and engineered elements (1 to 3). The engineering of these elements is indicated in (B) to (E), and their reference qB2H-responsive promoter—based on the OL2 cI binding element and L promoter −35 and −10 boxes—is indicated by a blue line (v1_OL2, -■-, light blue). (B) The promoter proposed by Hochschild and colleagues [9] (Hochschild; -•-, black) and an alternative version (Hochschild_OL2_lac2; -▽-) provide low signal and high stochasticity compared to v1_OL2. (C) Promoters comprising 2 cI binding elements result in poor or inverted *K*_d_ *vs.* signal correlations (-●- [black] v1_Ranganathan, pRM*: originally used by Ranganathan and colleagues [16] with functional OR1 and OR2 elements; -+- v1_Ranganathan_OR2-44: pRM* variant where OR2 was displaced at −44 from transcription start site (TSS); -⬟- v1_Ranganathan_OR2-62: *pRM* * variant where OR2 was displaced at −62 from TSS; -▼- v1_OL1-OL2-64: similar to v1_Ranganathan_OR2-62 but harboring OL1 and OL2 elements, the latter is displaced at −64 from TSS and −35 and −10 boxes derives from lambda L promoter). (D) Comparison of B2H-responsive promoters harboring only one of the natural cI binding elements (OR1, OR2, OR3, OL1, OL2, or OL3) at position −62 from the TSS of *L* using 2-plasmid system (v1). Intermediate-affinity elements result in higher signals. (E) Comparison of signal output (normalized mean fluorescence intensity [MFI]) from double (v1, ASF1A: -■- [light blue], ASF1B: -⬟- [black]) or single-plasmid series (v2, v3, and v4; -◆- [light green], -•- [dark green], and -▼- [red], respectively) on pOL2-62_L output. v1_OL2* is the same reference version as other panels. qB2H versions without “*” indicated that ASF1B_N_ was used instead of ASF1A_N_. Since v4 system do not harbor eGFP (Table 1), no fluorescence is observed. Single plasmids provide higher signal. The confidence interval (ci = 95%) through (B) to (E) was represented by semitransparent regions and results from 3 independent experiments (Table S3). (F) Comparison of the fluorescence of colonies transformed with the v3 plasmid series. Overnight cultures of SB39 VN1312 (left; ASF1B_N_–ip3mut3a interaction, *K*_d_ > 100 μM) and SB39 VN1315 (right; ASF1B_N_–ip3, *K*_d_ = 93 nM) grown on LB agar plates supplemented with chloramphenicol (34 μg/ml), IPTG (200 μM), and anhydrotetracycline (aTc; 200 ng/μl) were visualized using a blue light transilluminator (Safe Imager, Invitrogen, catalog no. G6600EU). Stronger fluorescence is observed for the stronger affinity.

v1, v2, v3, and v4 will be colored respectively in light blue, light green, dark green, and red, respectively, in Fig. 1 and Fig. S1.

B2H, bacterial two-hybrid; qB2H, quantitative B2H; RBS, ribosome binding site; T.I.R., translation initiation rate.

To guide the engineering of the qB2H-responsive promoter, we used a set of peptides with varying affinities [17] (ip3_mut3A, >100,000 nM; ip1, 8,700 nM; ip2, 500 nM; ip3, 55 nM; ip4, 3 nM; Table S1) for the N-terminal domain of ASF1A, ASF1A_N_ (UniProt: Q9Y294; positions 1 to 156). In our initial set of experiments, we used a 2-plasmid system (v1) to examine the effects of interactions on a set of 12 2H-responsive promoters (Table S2). The main goal was to explore 2 key features of the qB2H system: (a) the signal-to-noise ratio (ASF1–ip3 signal divided by ASF1–ip3_mut3A signal) and (b) the correlation between the signal output and the binding affinity (*K*_d_). We compared reported promoters to engineered alternatives by targeting specific functional regions. The promoters harbor 1 or 2 cI binding sites, with different binding affinities. Furthermore, the promoters included specific combinations of −35 and −10 boxes corresponding to different transcription strengths: *Lac* (weak), *RM,* and *L* (strong). A bicistronic construction allowing signal output as fluorescence and kanamycin resistance, eGFP-KanR, was chosen as reporter. A schematic representation of these elements is provided in Fig. 1A.

The weak promoter described by Hochschild and colleagues [9], along with OL2_lac2, shows reasonable coherence between signal intensities (mean fluorescence intensity [MFI]) and complex affinities (ip3_mut3A to ip4) but exhibits a low signal-to-noise ratio (2.36 to 3.23) and high variability (Fig. 1B). In contrast, promoters such as those proposed by Ranganathan and colleagues [16] (*pRM*), which contain 2 cI binding sites, provide an acceptable signal-to-noise ratio (6.73 to 10.95) but demonstrate poorer consistency with complex affinities (*K*_d_ *vs.* MFI coefficient of determination [*R*^2^] = 0.672 to 0.8) and even show an inverse correlation (v1_Ranganathan_OR2-62 *R*^2^ = 0.973; Fig. 1C and Table S3).

Next, we analyzed the system’s sensibility cI binding sites corresponding to decreasing affinities for λ cI: OL1 > OR1 > OL3 > OL2 > OR2 > OR3 [20,21]. Interestingly, the highest signals were observed for intermediate-affinity interactions (OL2 and OL3, respectively; 100 and 99.91 maximum normalized MFI), while the lowest- and the highest-affinity interactions produced the lowest signals, respectively: 29.44 and 78.02 (Fig. 1D). These results highlight the complex dynamics underlying B2H activity. Except for OR3, all cI binding elements demonstrate a strong correlation between *K*_d_ and signal for interactions ranging from tens of micromolar to nanomolar (*n* = 4, *R*^2^ between 0.935 and 0.999; Table S3), and undetectable interactions yield significantly lower signals than detectable weak interactions (*n* = 3 experiments, *P* < 0.05, *t* test; Table S3), allowing the latter to be distinguished from background. However, even if often not statistically significant, the signal intensities for the strongest interaction appear lower than the slightly weaker interaction. A possible explanation is that eGFP overexpression, which can lead to H_2_O_2_ accumulation [22] and increase metabolic burden [23], may result in counterselection against highly expressing clones—namely, those associated with high-affinity interactions.

A second set of experiments was conducted to improve the system’s quantitative properties. We merged all required B2H elements into a single plasmid (v2). We then focused on further improvements in signal-to-noise ratio and on reducing system stochasticity. To that end, we suppressed a methylation site (Dcm) in the qB2H-responsive promoter, simplified the B2H reporter gene, and sought to improve the *cI* and *rpoA* expression ratio and to reduce the required antibiotic concentration during selection experiments (v3). Finally, we explored the effect of ASF1B (UniProt: Q9NVP2), a paralog of ASF1A, on the signal-to-noise ratio.

Similar response profile and metrics were observed for single (v2_OL2) *vs.* 2-plasmid (v1_OL2) systems (signal-to-noise ratio = 18.77 to 19.92; *K*_d_ *vs.* MFI *R*^2^ = 0.954 to 0.971; coefficient of variation [CV] among experiments = 3.67% to 6.72%; Table S3) and for ASF1A_N_ *vs.* ASF1B_N_ (signal-to-noise ratio = 18.76 to 19.92; *K*_d_ *vs.* MFI *R*^2^ = 0.944 to 0.971; CV among experiments = 4.51% to 6.72%). Comparing Hochschild’s and Ranganathan’s promoters to the v3 series, the signal-to-noise ratio increases from 2.36 to 6.73 to 27.49 (highest signal presented in Fig. 1E and F), while the *K*_d_ *vs.* signal correlation improves from 0.783 to 0.786 to 0.952 (*n* = 4, *R*^2^; Table S3).

### Optimization for high-throughput experiments under antibiotic selection allows the identification of suitable conditions and data treatment options

Applying an appropriate selection pressure to a cell population, combined with NGS, allows estimation of genotype frequencies over time and calculation of their enrichment or depletion, which can be interpreted as fitness under the applied conditions. In this approach, antibiotics provide a convenient and effective means of imposing selection pressure.

**Fig. 2.**
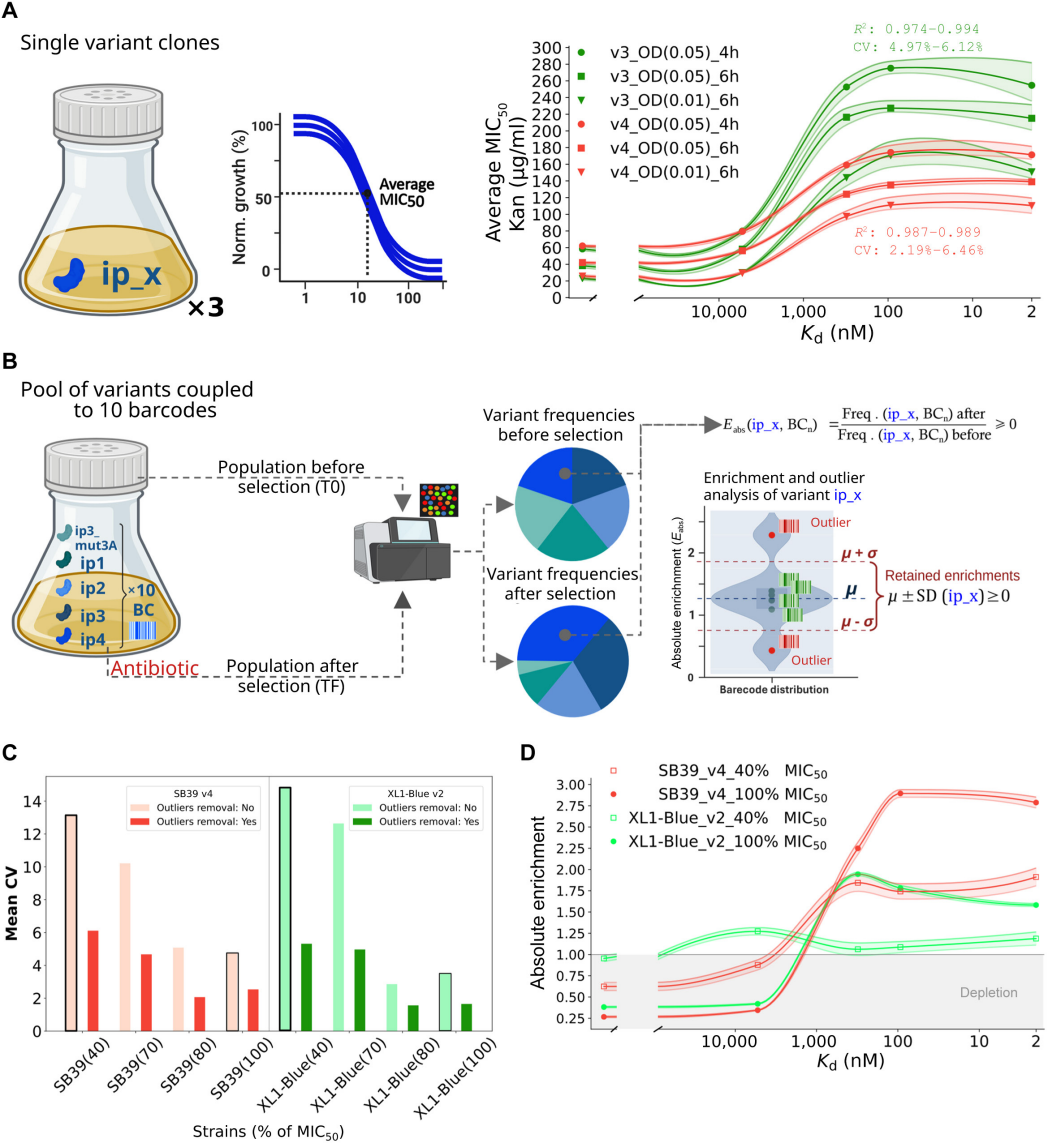
Effect of kanamycin-based selection on the growth of single and pooled variant clones. (A) Left: Schematic representation of the estimated average minimum inhibitory concentration at 50% (MIC_50_) for each interaction (ASF1–ip_x) at varying conditions. Right: Effect of *K*_d_ on antibiotic resistance (average MIC_50_). The experimental conditions are indicated by legend names composed of 3 fields separated by “_”, respectively: plasmid version, starting OD and time point (h) after kanamycin addition. The confidence interval (ci = 95%) was represented by semitransparent areas. The results represent 3 independent experiments. Triplicate MIC_50_ curves for each *K*_d_ are provided in the Supplementary Materials (Figs. S2 to S7 and Table S4). Lower ODs, longer time points, and v4 system are associated to relatively lower MIC_50_ values. (B) Comparison of nonoptimized and optimized systems. Schematic representation of the experiment designed to assess the behavior of optimized and unoptimized systems in pooled cocultures. Each peptide was coupled to 10 barcodes to estimate their independent frequencies and absolute enrichment (*E*_abs_), identify outliers and evaluate stochasticity (coefficient of variation [CV]). The right side of the panel represents calculation of *E*_abs_ for each peptide (ip_x) and barcode (BC_n_) from their frequencies before and after application of selection pressure (kanamycin supplementation). Enrichment values that are far from average (outliers, red) can be removed using *z*-score method to improve standard deviation (SD). (C) Mean CV by selection pressure (40%, 70%, 80%, and 100% of MIC_50_ for ASF1B_N_–ip3 interaction) for SB39 v4 (reddish) and XL1-Blue v2 (greenish) bacterial two-hybrid (B2H) systems. Light colors indicate no outliers’ removal. Conditions that are represented in (D) are surrounded by black lines. Higher selection pressures and outliers removal result in less stochastic data (CV). (D) Enrichment profile over the studied *K*_d_ range for SB39 and XL1-Blue strains under the lowest (40% MIC_50_) or highest (100% MIC_50_) selection pressure (without outliers’ removal). SB39 v4 provide better correlation between *K*_d_ and absolute enrichment. The experimental conditions are indicated by legend names composed of 3 fields separated by “_”, respectively: strain, plasmid version, and kanamycin concentration (as percentage of ASF1–ip3 MIC_50_). Enrichment of 1 (dashed gray line) corresponds to invariable frequency in the population before and after kanamycin selection. Raw data are available in the Supplementary Materials (Table S5).

Single-plasmid vectors linking PPI strength to kanamycin resistance—either as a single reporter (v4: KanR) or a dual reporter (v3: eGFP-KanR)—were compared in triplicate under different conditions. Minimum inhibitory concentration at 50% (MIC_50_) estimations (see “Methods”) were used to evaluate the effects of initial cell density (optical density at 600 nm [OD₆₀₀] = 0.05 or 0.01) and selection duration (4 or 6 h) on kanamycin resistance for each ASF1–peptide interaction (Fig. 2A, left). In the present work, MIC_50_ is defined as the antibiotic concentration that results in 50% growth inhibition of a given bacterial population in single-strain experiments.

Consistent with earlier studies [24,25], we observed lower MIC_50_ values (higher antibiotic sensitivity) at lower starting cell densities (OD₆₀₀ = 0.01) and longer selection times (6 h). These conditions also result in better signal-to-noise ratios (MIC_50_ ASF1–ip3/MIC_50_ ASF1–ip3_mut3A; ~65% increase). However, a starting OD₆₀₀ of 0.05 provided robust *K*_d_ *vs.* MIC_50_ correlations (*n* = 4, *K*_d_ values ranging from 10 μM to 2 nM, *R*^2^ ~ 0.99), as well as more reproducible results (mean CV = 2.19% to 5.5%; Table S4).

Across all experimental conditions, no statistically significant differences were observed for MIC_50_ values corresponding to *K*_d_ values of 93 and 2 nM, suggesting a plateau in the tens-of-nanomolar to nanomolar range. Despite this observation, compared to the single-gene reporter (v4), the bicistronic reporter (v3) exhibits a pronounced signal drop for the strongest affinity interaction (ASF1B–ip4), higher MIC_50_ values and wider confidence intervals, reflecting higher stochasticity (Fig. 2A, right). The lack of a signal drop at the highest-affinity interaction (ASF1–ip4; *K*_d_ = 2 nM) is likely due to the absence of the eGFP reporter and to dynamics of complex formation involving several interactions: cI-DNA, ASF1–ip4, and subunits of the bacterial RNA polymerase.

Consequently, owing to its superior *K*_d_ *vs.* MIC_50_ consistency and lower data variability, the v4 system is considered the most suitable for quantitative interaction analysis. Mapping these antibiotic sensitivity landscapes helped define the optimal antibiotic concentration range for subsequent stochasticity analyses and benchmarking of the optimized systems (Fig. 2B). To that end, we compared v2 in the XL1-Blue strain (the B2H chassis used in the commercial BacterioMatch II kit, Agilent) with the v4 series in SB39 strain. SB39 is a fast-growth *endA recA* derivative of BL21(DE3), created by the insertion of DNA cassette comprising 6 inducible repressors into its genome (full genotype provided in Table S11).

We focused on 2 key properties relevant to qB2H systems for PPI quantification: (a) the effect of selection pressure on stochasticity, using the CV as a proxy, and (b) the *K*_d_ *vs.* enrichment correlation. CVs were calculated as the percentage of enrichment standard deviation (from 10 unique DNA barcodes for each ASF1 peptide binder) compared to the mean enrichment. We also evaluated the effect of outliers’ removal on data discrepancy (Fig. 2B).

The results show that outliers’ removal using a *z*-score threshold considerably reduces data dispersion (mean CV) for both systems, particularly under conditions of low selection pressure (Fig. 2C). Furthermore, this approach does not markedly change mean enrichment values (Table S5). However, for selection pressures corresponding to 40% or 100% MIC_50_ of the ASF1B–ip3 interaction, the SB39 v4 showed better *K*_d_ *vs. e*nrichment coherence (worse *R*^2^ = 0.96, *n* = 4) than XL1-Blue v2 (worse *R*^2^ = 0.082, *n* = 4; Fig. 2B and Table S5). The results also indicate that higher selection pressures enhance the predictive linkage between enrichment and *K*_d_ and reduce stochasticity.

Taken together, we found that the SB39 strain, v4, and high selection pressure (70% to 150% MIC_50_ of a strong interaction for a given couple of partners) yield the best quantitative results.

### qB2H v4 supports protein interface-mapping strategies, enabling the identification of interaction hotspots and key contact residues

**Fig. 3.**
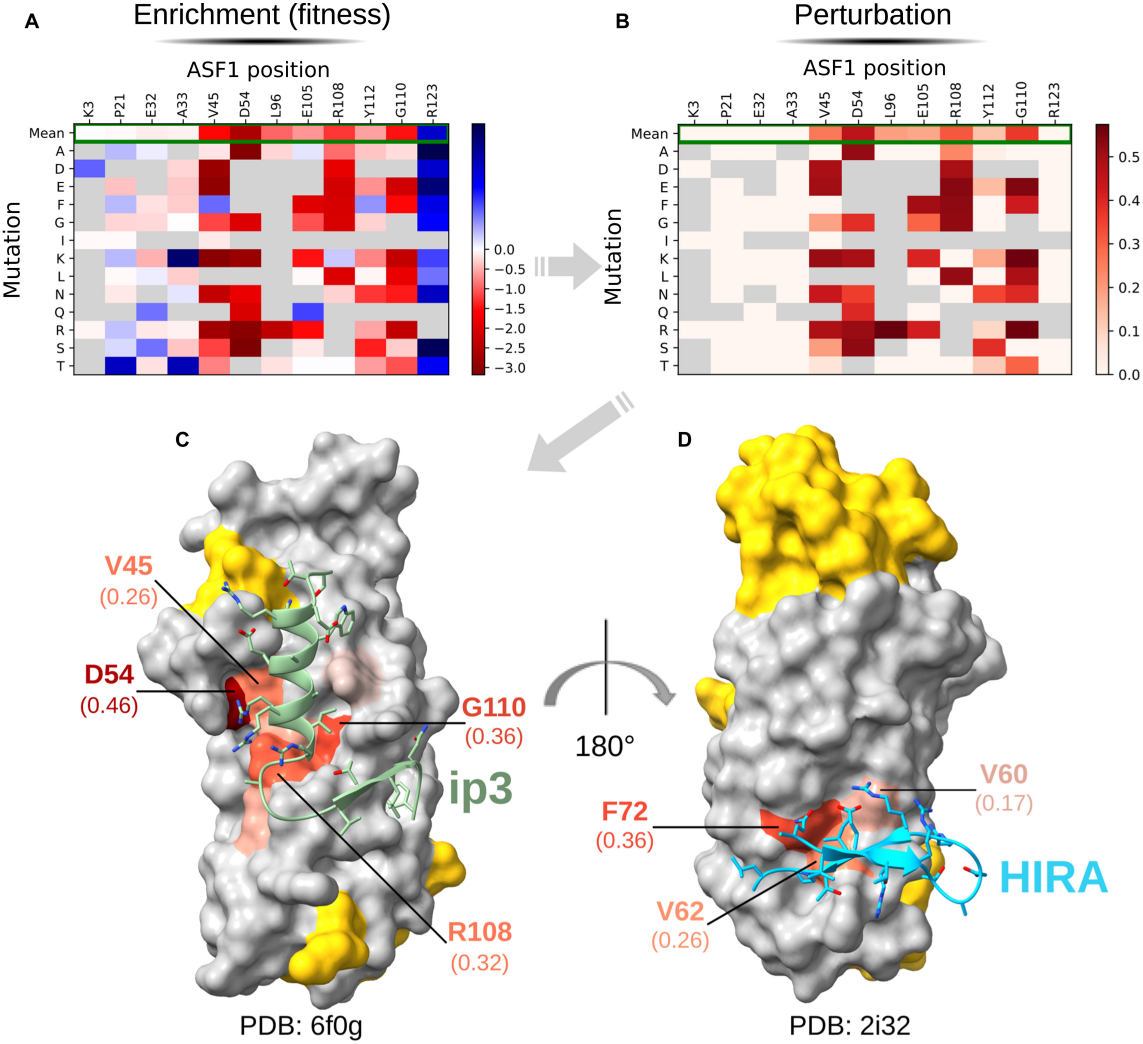
Demonstration of the interface mapping application. (A) Analysis of next-generation sequencing (NGS) data from the population before and after selection allows obtaining normalized enrichment for each variant (compared to reference sequence, wild type [WT]). To underline the interface mapping strategy, representative positions are displayed as a heatmap, colored on the basis of their log_2_-normalized enrichment. A white-to-red gradient represents loss of interaction, while a white-to-blue gradient represents gain of interaction. (B) Since mutation of interface residues are more likely to negatively affect interaction, enrichment scores are used to calculate perturbation scores (loss-of-interaction scores). Only representative positions are shown to illustrate the interface mapping logic [same as in (A)] as a heatmap colored on the basis of perturbation scores, using a white-to-red gradient. For clarity, only amino acid substitutions that (a) occurred in at least one of these representative positions and (b) had a nonzero fitness effect across all positions were retained in the display. (C) Interface mapping obtained for the ip3 binder (green; PDB: 6f0g). (D) Interface mapping obtained for the HIRA binder (light blue; PDB: 2i32). In (C) and (D), the top most perturbed positions (above a threshold = 0.1) are labeled with their names and perturbation scores (in parentheses) and mapped onto the target’s (ASF1) surface, colored as a gradient from light orange to red based on their perturbation scores. Nonperturbed positions are shown in gray. Some positions of the target were not assessed (yellow) since the exploration of 2 regions for each partner already held sufficient positions that could be assumed as perturbation controls (not found at the interface) to be compared to interface positions. High perturbation scores match critical interface contacts (hotspots).

Using the qB2H plasmids, we next evaluated the method’s reliability for mapping the interaction surface between ASF1 and binding partners whose complexes with ASF1 have been solved by x-ray crystallography. Our interface-mapping strategy (Fig. 3A and B) was applied to 2 different binders through independent experiments using qB2H v4: ip3 (Fig. 3C) and HIRA (histone regulatory homolog A) (Fig. 3D). Similar to ASF1, HIRA is a histone chaperone specialized in the replication-independent deposition of histone variant H3.3 onto chromatin. ASF1A directly interacts with HIRA on silenced chromatin domains and promotes histone deacetylation, contributing to heterochromatin formation [26,27].

Briefly, positional single-mutant libraries of ASF1 were designed under strategic and practical constraints. The corresponding DNA libraries were ordered from Twist Bioscience and cloned into the pB2H v4 series (details in “Methods”; “Construction and screening of interface mapping libraries” section in the Supplementary Materials; and Tables S1, S2, and S7). Cell libraries representing more than 100-fold coverage of the theoretical nucleotide diversity were compared before and after antibiotic selection by NGS (Illumina NovaSeq X). Data analysis with the mave2imap pipeline enabled variant count in these populations, the calculation of normalized enrichment values for each variant compared to wild-type (Fig. 3A, Table S8, and Fig. S9), and the assignment of a perturbation score to each assessed position (Fig. 3B, Table S9, and Fig. S10).

Since enrichment directly reflects binding strength under selection pressure (Fig. 2, v4) and mutations at interface positions are more likely to disrupt complex formation, interface residues can be inferred by identifying the positions associated with higher deleterious effects. Therefore, we define the perturbation score of a position as the average of the deleterious effects of its individual mutations (see the “Construction and testing of libraries for stochasticity assessment” in the Supplementary Materials).

The perturbation scores mapped onto ASF1’s structure overlaps with interface contacts observed in crystallographic complexes.

Overall, our results demonstrate the applicability of the qB2H v4 system for interface mapping, as well as for identifying interaction hotspots.

### Screening of binders using qB2H v4 yields improved variants in batch or turbidostat cultures

Artificial intelligence (AI)-based approaches, such as those implemented in BindCraft [28], represent major advances for *in silico* binder design and provide sets of solutions to be experimentally evaluated. We tested whether our qB2H v4 system can select an optimal solution among a set of generated models. Using a generative AI pipeline (coupling RFdiffusion [29], ProteinMPNN [30], and AlphaFold2 [31–33]), we generated and selected hundreds of N-terminal extensions of the ip4 peptide to be screened by using qB2H v4. These designs were coupled to 3 alternative linkers of variable lengths (GSK, GSEK, and GSEAK). This dataset also included a restricted number of manually designed variants based on structural inspection of the modeled interface RFdiffusion (so-called “rational designs”) and some negative controls. These variable extensions were fused to a constant peptide, ip4_mutG (WARLARRTAGAGGVTLNGAG), which was used as constant C-terminal scaffold. This peptide contains a mutation that lowers ip4 affinity for ASF1B_N_ (*K*_d_ = 10.6 μM), thereby enabling the selection of extensions that enhance interaction strength within the dynamic range of the qB2H system by exploring additional contacts with the ASF1’s surface.

**Fig. 4.**
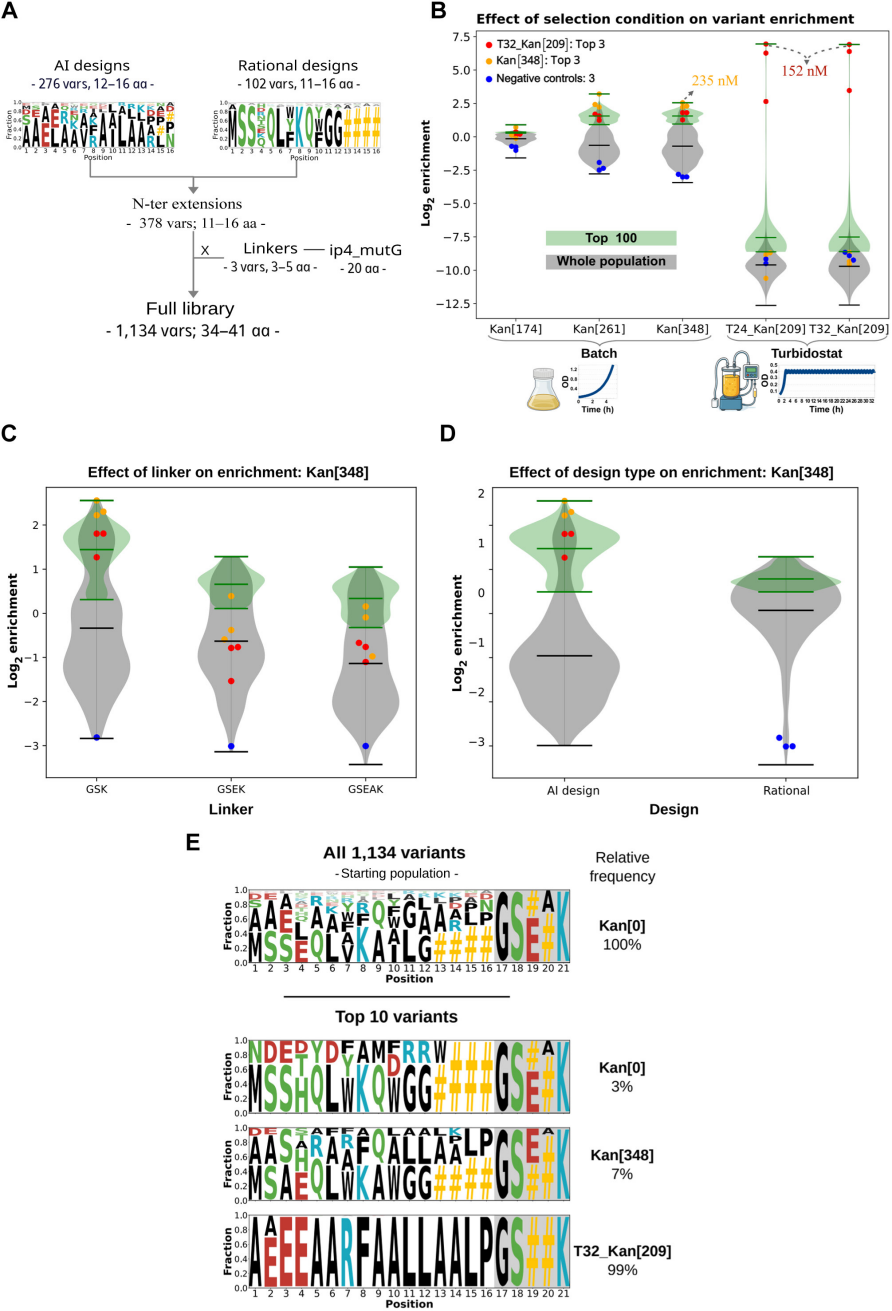
Binder selection experiment. (A) Library structure (N-terminal extension–linker–ip4_mutG), composition, and diversity. (B) Impact of selection condition on variant enrichment. Violin plots show the enrichment of all variants (gray). Since top-ranked candidates are those most likely to be experimentally pursued and to probe the effect of condition variables on this specific population, the top 100 variants (green) are also shown under different kanamycin concentrations in short-term (batch: Kan[174], Kan[261], and Kan[348]) or the long-term experiments (turbidostat: T24_Kan[209] and T32_Kan[209]). More stringent selection pressures result in stronger effects (enrichments or depletion). (C) Effect of linker on short-term (batch: Kan[348]) selection experiments. Smaller linkers tend to be more enriched. (D) Effect of design approach (AI or rational) on short-term (batch: Kan[348]) selection experiments. AI design results in a bimodal distribution, while rational design results in a single-mode distribution of enrichments. In (C) and (D), blue dots (●) correspond to negative controls (GSAGSAGSAGSAGS-(GSK/GSEK/GSEAK)-ip4_mutG), orange dots (●) correspond to the top 3 variants from batch Kan[348] (SAAARWAAALAALP-GSK-ip4_mutG, AAAERARFAALLAKLP-GSK-ip4_mutG, and SAAERFAAALAALP-GSK-ip4_mutG, respectively), and red dots (●) correspond to the top 3 variants from turbisdostat T32_Kan[209] (AEEEAARFAALLAALP-GSK-ip4_mutG, AAEEAARFAALLAALP-GSK-ip4_mutG, and AAEEQAAFDALLAALP-GSK-ip4_mutG, respectively). (E) WebLogo representing the fraction of amino acid (aa) residues by position for the entire population before selection (top of the WebLogo) and for the top 10 most frequent variants under different conditions (Kan[0], Kan[348], and T32_Kan[209]). Relative frequencies of the selected number of variants are shown (right) as percentage for each condition.

The resulting library encompasses 1,134 peptides composed by a variable and a constant region with global lengths ranging from 34 to 41 amino acids (Fig. 4A). The library was subjected to 2 different selection conditions: (a) short-term (4 to 5 h) batch selection using increasing kanamycin concentrations (0 and 174 μg/ml: 100% MIC_50_, 261 μg/ml: 150% MIC_50_, and 348 μg/ml: 200% MIC_50_ of ASF1–ip3 interaction) and (b) long-term (24 and 32 h) selection in a turbidostat with fixed kanamycin concentration (209 μg/ml: 120% MIC_50_ of ASF1–ip3 interaction). Samples collected before (*T*_0_) and after selection (*T*_F_) were compared to evaluate the impact of different selection pressures on population outcomes.

We selected 734 variants present in all short- and long-term samples to calculate the Pearson correlation coefficient (*r*), which measures the degree of similarity in phenotypic behavior between pairs of samples. As expected, stronger selection pressures lead to greater frequency shifts (enrichment or depletion), reflecting the relative fitness of each variant. In batch experiments, variant frequencies under kanamycin (174 μg/ml) correlated better with the nonselected population (*r* = 0.96, *P* = 0) than with those under stronger selection pressures: 261 μg/ml (*r* = 0.76, *P* = 3.03 × 10^−163^) or 348 μg/ml (*r* = 0.76, *P* = 1 × 10^−162^). Selection at 261 and 348 μg/ml produced nearly identical frequency profiles (*r* = 0.99, *P* = 0), indicating that increasing the concentration from 150% to 200% of the ASF1B_N_–ip3 MIC_50_ did not further affect variant frequencies within the time range. In long-term turbidostat experiments (24 or 32 h at constant OD), variant frequencies were more correlated to those observed at higher kanamycin concentrations in batch culture than to those under lower or no selection. Moreover, continuous cultures at 24 and 32 h displayed almost identical profiles (*r* = 0.99, *P* = 0), indicating that 24 h of selection is sufficient to produce strong shifts in variant frequencies compared to no selection (*r* = 0.24, *P* < 3.12 × 10^−11^). High correlation coefficients (*r*) across similar conditions serve as pseudo-replicates, confirming the system’s reproducibility over large libraries. A detailed comparison among conditions is provided in the Supplementary Materials (Figs. S11 to S22 and Table S10). Figures S17 and S18 provide a general overview of the phylogenetic relationships and fitness landscape of the screened library.

These results suggest that 4 to 5 h of selection at 150% of the MIC₅₀ of a strong interaction provides a suitable starting point for short-term batch experiments, while 24 h of selection at 120% MIC₅₀ can be used for long-term experiments. In long-term experiments, the top 2 enriched variants accounted for about 89% of the final population (66% and 23%; Table S10), showing strong and reproducible convergence toward closely related N-terminal extensions differing by only one amino acid substitution and sharing the same linker sequence (GSK). Under these conditions, the bulk of remaining variants show a pronounced drop in frequency, with many approaching zero or disappearing entirely. For milder selection in turbidostat, lower kanamycin concentration should be used. Figure 4B illustrates the effect of increasingly stringent selection conditions on binders’ enrichment. Overall, the results show that stronger selection conditions produce more pronounced enrichment or depletion effects and that the turbidostat selection represents the harshest tested condition.

The impact of the linker sequence can also be assessed from the results. The short linker (GSK) is more enriched than longer ones (Fig. 4C). Specifically, under the most stringent selection condition (T32_Kan[209]; Table S10), GSK, GSEK, and GSEAK account for 66%, 21%, and 13%, respectively, of the top 100 most frequent variants. Moreover, the GSK linker corresponds to 9 of 10 most frequent variants. This effect is probably related to the reduction of entropic cost for shorter linkers.

Across the full set of variants, the so-called “rational designs” showed higher average enrichment than AI designs. However, this trend was reversed considering only the top 100 variants (Fig. 4D). Overall, under the present experimental conditions, AI design produced a small fraction of high-quality solutions amid most low-quality variants, while rational design generated a distribution centered on intermediate-quality solutions.

The effect of different selection conditions (no selection to Kan[0], Kan[348], or T32_Kan[209]) on residue frequencies by position, as well as the frequencies of the top 10 variants relative to the entire population, reveals that the diversity of variants in the population decreases with more stringent selection conditions (Fig. 4E).

### The top-ranked binders are validated by ITC and nuclear magnetic resonance

The top 2 variants from the most stringent condition in batch and turbidostat selections were considered for production and evaluation by ITC. T32_Kan[209]_top2 was not kept in the list, as it differed by only one amino acid from T32_Kan[209]_top1. The 3 retained variants, T32_Kan[209]_top1 (AEEEAARFAALLAALP-GSK-ip4_mutG), Kan[348]_top1 (SAAARWAAALAALP-GSK-ip4_mutG), and Kan[348]_top2 (AAAERARFAALLAKLP-GSK-ip4_mutG), all holding the GSK linker, were expressed and purified, and their affinities for ASF1A_N_ were measured using ITC (Fig. S23).

The best binder, T32_Kan[209]_top1, showed an affinity of 152 ± 25 nM corresponding to a 70-fold improvement compared to ip4_mutG (*K*_d_ = 10.6 ± 1.0 μM), followed by Kan[348]_top1 (235 ± 26 nM, 50-fold improvement) and Kan[348]_top2 (581 ± 98 nM, 20-fold improvement). Remarkably, the measured affinities correlated with their selection stringency and enrichment levels.

**Fig. 5.**
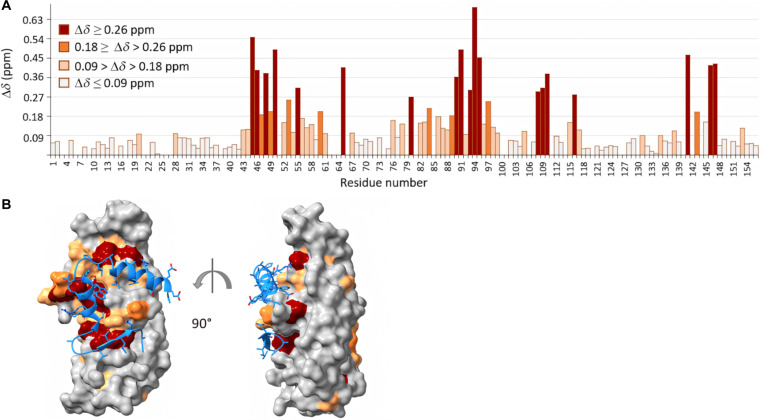
ASF1A_N_–T32_Kan[209] binding mode. (A) Nuclear magnetic resonance (NMR) titration of ^15^N-ASF1A_N_: chemical shift perturbation observed after the addition of an excess of T32_Kan[209]_top1 peptide (^15^N-ASF1A_N_: T32_Kan[209]_top1 ratio = 1:2). Chemical shift changes of residues are highlighted with the following color code: dark red (Δ*δ* ≥ 0.26 parts per million [ppm]), dark orange (medium: 0.18 ≥ Δ*δ* > 0.26 ppm), light orange (low: 0.09 > Δ*δ* > 0.18 ppm), and gray (background: Δ*δ* ≤ 0.09 ppm). (B) AlphaFold3 model of ASF1A_N_ bound to T32_Kan[209]_top1. The optimized peptide is shown as a cartoon and colored in blue. ASF1A_N_ surface is colored as in (A). Upon binding, higher chemical shifts perturbations match ASF1 positions at the interface predicted by AlphaFold3.

We next analyzed the binding mode of the best binder, T32_Kan[209]_top1, with ASF1 using nuclear magnetic resonance (NMR) spectroscopy. Chemical shift perturbation of uniformly labeled ASF1A_N_ upon addition of unlabeled T32_Kan[209]_top1 was measured and then mapped on the AlphaFold3 model of the corresponding complex (Fig. 5). The surfaces interacting with both the initial ip4_mutG peptide and the designed N-terminal extension are highlighted, showing that the chemical shift changes are in good agreement with the predicted binding interface.

## Discussion

Building from previously described B2H systems based on λ *cI* and *Escherichia coli rpoA* fusions engineered by A. Hochschild (Harvard University), R. Ranganathan (University of Chicago), and their colleagues, we engineered optimized variants with improved quantitative properties (qB2H). In early versions of Hochschild’s B2H, the PPI responsive promoter had an OR2 cI-binding element centered at position −62 from transcription start (*Lac* OR2-62) inserted into the genome of KS1 strain (MC1000 F′ *lacI*^q^ with *Lac* OR2-62 *lacZ*) [9], while later versions used the OL2 element at the same position (*Lac* OL2-62) inserted into the F episome of FW102 strain [34]. Our results corroborate that the OL2 cI binding element is indeed a better option with regard to quantitative metrics. Ranganathan and colleagues [16] proposed an alternative implementation in 3 plasmids (pZA31-RNAα, pZS22, and pZE1RM-eGFP) that used MC4100 Z1 strain as chassis (MC4100 cells that express LacI [lactose] and TetR [tetracycline] repressors). pZA31-RNAα was designed to express *rpoA* fusions, pZS22 to express cIE34P fusions and pZE1RM-eGFP to provide an eGFP-based reporter under control of a λ *pRM* promoter carrying 2 λ cI binding sites and a C-to-T mutation at the 10th position of the OR3 element, which abolishes cI binding and provides stronger output [35]. Our results show that the presence of the 2 binding sites negatively affects the correlation between *K*_d_ and signal.

Based on these system features, we anticipated incompatibilities with accurate quantification of PPIs, which were confirmed by the results and are expected to negatively impact the generation of high-quality datasets. Machine learning models have been increasingly used in diverse fields, including protein engineering, where data quality is well recognized as crucial for model training, interpretation, and application.

Since most *E. coli* strains express lac repressor (LacI), cotransformation with v1 (pAC-cI_E34P and pBRa) or transformation with v2 plasmid series allows for inducible and strain independent selection based on both fluorescence (using fluorescence-activated cell sorting) and/or kanamycin resistance. However, since the KanR gene expression was not optimized in these systems, the required kanamycin concentration can be remarkably high for strong interactions, especially for v2 plasmid series (e.g., ≥1 mg/ml for interactions such as ASF1B–ip3). The v3 plasmid series cumulates additional modifications that lead to higher signal-to-noise ratio, lower antibiotic concentration requirement, and reduced experimental stochasticity. These modifications include the invalidation of Dcm site close to the −35 box of the qB2H-responsive promoter and a stringent control of l cI_E34P fusions from a strong and tightly regulated promoter (LTetO promoter) [36] coupled to a weak ribosome binding site (RBS). Indeed, earlier work demonstrated that this kind of setup can reduce stochasticity [37]. Single plasmid from v4 series incorporate all the latter features, but they were also engineered to provide lower and monocistronic expression of KanR that reduce the system’s complexity and improves quantitative measures. qB2H v1 to v3 support fluorescence-based screening (Fig. 1B to E) via fluorescence-activated cell sorting or colony visualization on agar plates, while all versions (v1 to v4) support kanamycin-resistance-based screening in liquid or solid medium. If v3 and v4 are used in strains lacking TetR, cI will be constitutively expressed. These properties can be exploited when sequencing resources are limited or rapid screening is needed.

The qB2H systems presented here demonstrate high-quality quantitative metrics, indicating reliable assay performance. The latest dual reporter version, v3, provides a high signal-to-noise ratio (27.49 for the fluorescent reporter), *K*_d_–signal consistency (*R*^2^ = 0.952), and low interexperiment variation on (CV = 7.04% for fluorescent reporter and 4.97% to 6.12% for kanamycin resistance reporter) in SB39 strain. pB2H v4 was derived from v3 by replacing the dual reporter with a single antibiotic resistance marker and shows slightly improved metrics such as high *K*_d_–signal consistency (*R*^2^ *=* 0.99), reproducibility (Pearson correlation of pseudo-replicates, *r* = 0.99, *P* = 0, *n* = 734), and low interexperiment variation (CV = 2.19% to 6.46%). Quantitative fitness values can be derived from NGS data, and barcode implementation provides statistical robustness and enables identification of outlier clones. Concerning the removal of outliers, narrower *z*-scores around the mean (e.g., 0.5 or 0.25) can further reduce standard deviation. This property can be advantageous to improve statistics for applications that rely on it, such as perturbation score calculations (see the “Interface mapping” section) and epistasis analysis using AI [38].

Very low affinities can be detected using qB2H, as demonstrated by the successful detection of interactions between suppressor of mif two 3 (SMT3) and peptides bearing small ubiquitin-like modifier (SUMO)-interacting motifs, whose affinities are often reported in the range of 10 to 100 μM (Fig. S25). This capability can be exploited in peptide and protein binder optimization campaigns starting from low-affinity scaffolds, which can be extended to establish new contacts aimed at improved affinity, as shown here.

Regarding general system features, the results indicate that higher selection pressures enhance the *K*_d_ *vs.* enrichment correlation and reduce experimental stochasticity. Nevertheless, very stringent selection pressures, such as those achievable in turbidostat experiments, can drastically impact population outcomes, leading to the large dominance of high-affinity binders and the extinction of low-affinity ones. In other words, long-term selection drives extreme enrichment and depletion, resulting in convergence of the population toward high fitness phenotypes. This strategy can be useful for isolating high-affinity binders but would be injudicious when trying to generate comprehensive datasets, for which short-term experiments are advisable.

We demonstrated here that qB2H can be used for epitope mapping. Concerning ASF1–ip3 interface mapping results, we observed that mutations at position R123 frequently result in higher enrichment compared to the wild-type sequence (Fig. 3A and Table S8). Comparing the ASF1 apo (Protein Data Bank [PDB]: 7LNY) and ip3-bound (PDB: 6F0G) structures, the helical segment at R123 shifts slightly upon binding. In the apo structure, the R123 NH1 atom hydrogen bonds with the Y117 backbone oxygen (R123 average heavy-atom *B*-factor ≈ 91), while in the complex, this contact is replaced by an NH1–water hydrogen bond (average heavy-atom *B*-factor ≈ 60). We hypothesize that this forced rotamer switch imposes a conformational penalty on binding and that nearly any substitution at R123 removes this barrier and improves affinity.

The comparison of generative AI-based and rational designs—the latter derived from crystallographic complexes—in the binder screening experiment reveals a striking difference in affinity distributions: AI-based approaches yield a bimodal distribution with a small but notable fraction of high-affinity binders, whereas rational design produces a unimodal distribution centered on moderate affinity. This observation aligns with the broader notion that AI methods explore a wider, less constrained sequence and conformational space, occasionally identifying high-affinity solutions beyond the reach of human intuition [39–41], while rational design—anchored to a validated structural starting point—yields more predictable but less diverse outcomes [42,43].

In the context of validated PPIs, the assay can also be exploited to study the impact of point mutations involved in disease-associated phenotypes and pathogen–host relationships. Another application would be the engineering of complex interfaces to allow orthogonal cell signaling rewiring and the conversion of homodimeric interfaces into heterodimeric ones. We are currently investigating the latter application for the selection of heterodimeric immunoglobulin domains to improve bispecific antibody production by supporting arbitrary heavy–heavy and heavy–light chain pairing.

Despite its strengths, the qB2H also has some limitations. For instance, signal output can be impacted by marked changes in fusion expression, and rank order may shift for variants with similar *K*_d_ values if the assay conditions are not stringent enough to discriminate between them. In the latter case, barcodes can be used to track individual clones exhibiting outlier phenotypes—a strategy also valuable for identifying escape mutants that could evade selection pressure through unexpected mutations. In addition, subcloning of selected variants into a fresh plasmid (v3) followed by clonal evaluation using flow cytometry can serve as a fast intermediate validation step prior to in-depth biophysical characterization of binding affinity. Since the system seems to achieve a plateau in the nanomolar range, it is not suitable for evaluating affinities in the picomolar range, which constitutes an additional limitation. Furthermore, evaluating PPIs involving bacterial proteins may suffer from interference by host cell proteins, generating false-positive and false-negative readouts [44].

The qB2H proposed here can be compared to other systems. An interesting cell-free 2H alternative was recently proposed by Bonnet’s group [45]. This system provides a fast way to evaluate individual PPIs at medium throughput (dozens to hundreds per day) but is limited by the impact of fusion expression level, solubility, and stability and does not provide the iterative, fitness-coupled selection pressure required for competitive screening of large variant libraries. By adding an abundance evaluation experiment to the dihydrofolate reductase (DHFR)-based yeast PCA assay, deepPCA (developed by Lehner’s and Diss’ laboratories [46,47]) provides a means to assess the expression levels of variants and evaluate their impact on DMS results [38]. It requires, however, running 2 independent experiments. While highly quantitative, this yeast-based approach requires more complex transformation protocols, longer generation times, and a eukaryotic cellular environment where endogenous proteins may interfere with the studied interaction due to phylogenetic relationship. Deep mutational scanning workflows in yeast also require careful optimization of harvest timing and library composition to avoid nonlinearities [47]. The qB2H system combines growth-coupled competitive selection, low-cost bacterial infrastructure, and quantitative affinity readout in a single experimental workflow, making it particularly well suited for high-throughput variant library screening. Fusing fluorescent proteins, such as eGFP and red fluorescent protein, to qB2H v4 could allow a similar abundance evaluation using a single library and a more convenient protocol.

A similar qB2H system was recently reported by Kosuri and colleagues [48] relying on cyclic adenosine monophosphate (cAMP) overproduction as a function of the PPI affinity. While this represents a substantial advance toward a qB2H, the cAMP receptor protein (CRP)–cAMP complex directly regulates approximately 200 genes in *E. coli* by binding with high affinity [49,50], with over 100 of these genes involved in metabolic pathways for alternative carbon source [51]. Computational analyses further identify more than 10,000 lower-affinity sites, indicating broader effects mediated by weaker interactions [50]. Such nontargeted effects may impose unnecessary burdens and potentially result in stochastic consequences in specific cases. The growth medium, especially glucose concentration, modulates intracellular cAMP levels and thus influences B2H assay outcomes. The reporter used, superfolder green fluorescent protein (sfGFP), may result in counterselection against high-affinity clones, as observed here. Unfortunately, we could not find clear data correlating *K*_d_ values with signal intensity. Although the authors claimed plasmids were deposited at Addgene, we could not locate them, limiting adoption and reproducibility in the research community.

In contrast, the specific interaction between λ cI and its cognate DNA sequence is expected to enhance insulation of the synthetic system, thereby minimizing off-target effects and improving robustness across laboratories. In the case of the qB2H system described here, all necessary materials have been made publicly available through Addgene. The SB39 strain represents the convergence of some key features that make it very convenient for qB2H experiments: (a) fast growth; (b) presence of chromosome gene copy of some of the most used transcription repressors (*phlF*^AM^, *cymR*^AM^, *luxR*, *vanR*^AM^, *lacI*^*A*M^, and *tetR*) for genetic circuit design and regulation (suitable for future engineering of biological circuits); (c) deficiency in Lon and OmpT proteases that improves protein stability; (d) *endA* and *recA* mutations that improve plasmid transformation and stability, making it compliant with library construction and screening. Furthermore, this strain shows elevated electroporation efficiency (>1 × 10^9^ colony-forming units/μg of pUC19) using in-house preparations [52].

While direct benchmarking against externally characterized libraries and the demonstration of additional applications was beyond the scope of this foundational study, future work will leverage such comparisons to further establish the assay’s performance relative to existing high-throughput approaches. Currently, promising results arise from preliminary data regarding the generation of high-quality antibody–antigen structural models using AI tools (such as Chai-1 and Boltz-2 [53,54]) based on dpDMS. AI-based structure predictions often provide high-quality models of individual subunits, but models of complexes can suffer from low interface confidence. This is especially true when coevolutionary information is scarce [55,56]. In such cases, the interface can be verified using single-partner DMS focusing on the associated positions. Expanding dpDMScan offer reliable constraints to guide the modeling of challenging complexes, such as antigen–antibody and host–pathogen interactions [55,57], by integrating experimentally informed generative AI approaches [53].

## Conclusion

Building on the current qB2H system, we anticipate that it will serve as a versatile platform for a wide range of projects. Table S12 and Fig. S25 demonstrate the general applicability of qB2H versions by evaluating additional model interactions, protein folding and quaternary structure, cell chassis, and induction conditions. Encoding both interaction partners on a single plasmid (pB2H v2 to v4) makes the system well suited for dpDMS analyses—a method with few reliable, user-friendly solutions—thus broadening access to this approach within scientific and engineering communities (see “Data Availability”).

Different applications are envisioned for qB2H, even beyond those demonstrated in this work. These include, but are not limited to, PPI discovery and validation, screening of AI generated binders, interface mapping, identification of binding hotspots, engineering of protein complex interfaces, high-throughput binder optimization, data generation for AI-driven studies, and 3-dimensional modeling of protein complexes constrained by experimental data. The motivation behind these applications may relate to understanding fundamental aspects of biology, physiopathological states, and microbe–host interactions.

The future directions, outlined by our ongoing projects, aim to address these applications, and preliminary data already provide supporting evidence. For instance, high-quality AI-based structural models of antigen–antibody complexes were obtained, and a dataset representing this kind of interaction was used to fine-tune the Evolutionary Context-integrated neural Network (ECNet) model [1], yielding a Spearman correlation coefficient of 0.93—further reinforcing the quality of the data generated by qB2H assay. Beyond our own investigations, we expect that these and yet unforeseen applications will be explored by other laboratories, further expanding the impact of the qB2H framework.

## Methods

### Strains, construction of plasmids, and precultures

XL1-Blue chemically competent cells were used for routine cloning. Marionette Clo, XL1-Blue, and SB39 strains were used as chassis for the experiments (detailed strain related information available in Table S11; SB39 strain is available at Addgene, ID: 235121). The SB39 strain was engineered using a bacterial implementation of Dual Integrase Cassette Exchange [58], achieved by inserting 6 repressors into the *glvC* locus of an *endA recA* derivative of BL21(DE3).

Detailed information about the oligonucleotides and the construction of vectors used in this work is available as supporting information (plasmid construction, Table S6: oligonucleotides, Table S2: plasmids, and Fig. S1).

Briefly, plasmids related to the 2-plasmids system were derived from pBRα and pACλcI–β-flap, engineered by Hochschild and colleagues (Harvard University) [59]. The latter, pACλcI–β-flap, was modified to include a B2H bicistronic reporter comprising eGFP and a kanamycin resistance coding region and to create a cI_E34P (GenBank: AAA96581.1; residues 1 to 237) fusion with the N-terminal domain of human ASF1A, ASF1A_N_ (UniProt: Q9Y294; residues 1 to 156), or the N-terminal domain of human ASF1B, ASF1B_N_ (UniProt: Q9NVP2; residues 1 to 156), giving rise to pAC-cI_E34P plasmid series. pBRα plasmid series correspond to the expression of 5 peptides (corresponding to different affinities against ASF1; Table S1) fused to the α subunit of *E. coli* RNA polymerase (RpoA lacking 81 C-terminal residues: residues 1 to 248; GenBank: AAC76320.1). The 2-plasmid system from v1 series was composed of one plasmid of pBRα and one plasmid of pAC-cI_E34P series.

Single plasmids from v2 series were created by assembling all the required B2H elements (from pAC-cI_E34P and pBRα series) into a single plasmid. Single plasmids from v3 series derived from v2 by invalidating a Dcm site in B2H-responsive promoter (*pOL2-62_L*) to reduce stochasticity and by adjusting the cI fusion expression using a strong inducible promoter combined with a weak RBS to maximize signal output and increased dynamic range. Finally, plasmids from v4 series were derived from v3 series by converting the bicistronic reporter into a single-protein reporter (KanR), optimizing its RBS strength through a weak RBS library and sequencing small colonies in inducing LB Agar supplemented with kanamycin (100 μg/ml).

Unless otherwise stated, precultures were inoculated late afternoon and grown overnight (37 °C at 190 rpm) from glycerol stocks in 10 ml of LB supplemented with antibiotics as required. The following final concentrations were used: ampicillin (100 μg/ml for single-plasmid selection and 75 μg/ml for 2-plasmid setups) and chloramphenicol (34 μg/ml for single plasmid and 25 μg/ml for 2-plasmid setups), anhydrotetracycline (aTc) (200 ng/μl for single-plasmid setups from the v3 and v4 series), and isopropyl-β-d-thiogalactopyranoside (IPTG) (20 nM for 2 plasmids from v1 and single plasmid from v2 series and 200 μM for single plasmid from v3 and v4 series).

### qB2H experiments using a fluorescent reporter

Precultures of untransformed (for intrinsic fluorescence evaluation) and transformed SB39 cells were diluted (3 μl into 300 μl of fresh medium) in 2-ml microtubes and grown in ThermoMixer C (Eppendorf; 37 °C at 900 rpm) for 2 h. Cultures with an OD_600_ of approximately 0.5 were then mixed with 100 μl of the same medium containing 4× inducer concentration. Final inducer concentrations, chosen on the basis of maximal signal output, were 20 μM IPTG for v1 or v2 and 200 μM IPTG plus 200 μM aTc for v3 series. The induced cultures were grown overnight at 18 °C, diluted (1 μl) in freshly filtered (0.22 μm) phosphate-buffered saline buffer (1 ml per OD unit), and analyzed using guava easyCyte HT (488-nm blue laser). The gating for event analysis was set to minimize background noise (fewer than 100 gated events per 10 μl of sterile phosphate-buffered saline). Ten thousand events were collected per culture, and the MFI was recorded. To allow comparison across independent experiments, SB39 cells cotransformed with VN519 (pBRα-*rpoA*-*ip3_mut3A*) and VN550 (pAC-cI_E34P-ASF1A_Term_f1-pOL2-62_L-eGFP-KanR) or VN517 (pBRα-*rpoA*-*ip3*) and VN550 were included as internal controls. Their MFI were used to normalize those of other conditions (*x*) using the following equation [Eq. (1)]:

$$\text{Normalized MFI}\left(x\right)=\left(\frac{\mathrm{MFI}\left(x\right)-\mathrm{MFI}\left(\mathrm{low}\right)}{\mathrm{MFI}\left(\text{high}\right)-\mathrm{MFI}\left(\mathrm{low}\right)}\right)\;\times \;100$$ (1)

MFI(*x*) represents the MFI of a given condition or culture. MFI(low) corresponds to the fluorescence produced by SB39 cells cotransformed with VN519 and VN550, indicating no detectable interaction (*K*_d_ > 100,000 nM). In contrast, MFI(high) corresponds to the fluorescence generated by SB39 cells cotransformed with VN517 and VN550, indicating a strong interaction (*K*_d_ = 55 nM). The signal-to-noise ratio was calculated by dividing the ASF1–ip3 MFI (high signal) by the ASF1–ip3_mut3A MFI (noise). Each experiment was conducted in 3 independent assays performed on different days. Figures were generated using Python notebooks in conjunction with Matplotlib.

### MIC_50_ estimations

Precultures of transformed SB39 cells were diluted in 50-ml tubes (50 μl in 5 ml of fresh medium supplemented with chloramphenicol [17.5 μg/ml]) and grown in an orbital shaker (37 °C at 190 rpm). After 2 h, cultures (OD_600_ ~ 0.5) were adjusted to OD_600_ = 0.05 in the same medium containing inducers: 20 μM IPTG (v2 series) or 200 μM IPTG plus aTc (200 ng/ml; v3 or v4 series). Cultures were incubated at 37 °C for an additional 2 h. Subsequently, 300 μl of culture at OD_600_ = 0.1 or 0.02 was prepared in induced medium and mixed with 300 μl of the same medium supplemented with varying 2× concentrations of kanamycin. Cultures were incubated in a ThermoMixer C (Eppendorf; 37 °C at 950 rpm), and OD_600_ was measured after 4 and 6 h. The MIC_50_ for each condition (including strain, plasmid, starting OD_600_, and time in the presence of kanamycin) was calculated by fitting the values of kanamycin concentration and the normalized OD (OD_600_ normalized to the condition without antibiotics, assumed as 100% growth). The signal-to-noise ratio was calculated by dividing the ASF1–ip3 MIC_50_ (high signal) by the ASF1–ip3_mut3A MIC_50_ (noise).

Python code for these calculations is available as a notebook. Each experiment was conducted in triplicate, with each replicate performed on a different day.

### Comparison of XL1-Blue v2 and SB39 v4

A detailed description of the construction and screening of interface mapping libraries is provided in the Supplementary Materials (“Construction and testing of libraries for stochasticity assessment” section and Tables S2 and S6). Briefly, DNA fragments encoding each ASF1 binder peptide, each coupled to 10 distinct barcodes, were generated and cloned into v2 or v4 series plasmids. Sublibraries, each corresponding to a specific peptide, were used to transform XL1-Blue or SB39 cells, respectively. Equiproportional cell libraries comprising all barcoded peptides were then prepared. Enrichment and stochasticity for XL1-Blue v2 and SB39 v4, as well as for each peptide, were evaluated by NGS under selection pressures corresponding to 40%, 70%, 80%, or 100% MIC_50_ of the ASF1–ip3 interaction within each system (strain plus plasmid series).

The CV, expressed as the percentage of the standard deviation relative to the mean enrichment, was used as a proxy to assess stochasticity.

### Interface mapping

A detailed description of the construction and screening of interface mapping libraries is provided in the Supplementary Materials (“Construction and screening of interface mapping libraries” section, Tables S2 and S6, and Fig. S8). Briefly, independent position libraries of ASF1B_N_ were obtained from Twist Bioscience, and equimolar pools of positions were cloned into the v4 series plasmid containing *rpoA*–*ip3* or *rpoA*–*HIRA* fusions. HIRA corresponds to residues 442–472 (UniProt: P54198), harboring an arbitrary point mutation, C465S, to prevent intermolecular disulfide bond formation. Both ASF1B_N_ binders were chosen because of the availability of crystallographic structures in complex with ASF1 (PDB: 6F0G and 2I32, respectively). SB39 cells were transformed with ASF1 region-specific libraries corresponding to 3 distinct position pools (N terminus, middle, or C terminus), and overnight precultures were induced. Cell populations were analyzed before (*T*_0_) and after antibiotic selection (*T*_F_) to assess the impact of each position on binder interaction by NGS coupled with data analysis.

The results were processed using the mave2imap pipeline and Python notebooks to generate fitness and perturbation score datasets and figures (see “Data Availability”). Briefly, the enrichment of a given variant ($E_{i}$) was calculated as described in Eq. (2) using the median enrichment of wild-type sequences [$\tilde{E}\mathrm{wt}$; Eq. (3)] for normalization.

$$E_{i}=c_{i}\;\text{after }\times \;\left(\frac{1}{\tilde{E}\mathrm{wt}}\right)÷\;c_{i}\;\text{before}$$ (2)

$$\mathrm{Ew}t_{x}=\frac{\mathrm{cw}t_{x}\;\text{after}}{\mathrm{cw}t_{x}\text{before}}$$ (3)

where $\mathrm{Ew}t_{x}$ is the enrichment of a wild-type or synonymous variant (x), $\mathrm{cw}t_{x}\;\text{after}$ is the counts of that variant after antibiotic selection and $\mathrm{cw}t_{x}\text{before}$ is the counts of the same variant before antibiotic selection.

Although other data analysis strategies have been developed [60] and can be used in compatible setups, the strategy described here included dephasers (to improve base diversity and sequencing quality across low-complexity libraries), unique molecule identifiers (for polymerase chain reaction (PCR) deduplication, error correction, and accurate variant quantification), and sequencing of independent regions of the same protein that must be assembled before reporting the final results (Supplementary Materials and Fig. S8), therefore justifying the development of mave2imap.

### Design and selection of binders

An AI pipeline integrating RFdiffusion, ProteinMPNN, and AlphaFold2 (detailed in Supplementary Methods) was implemented to design N-terminal extensions of ip4_mutG, a weak ASF1 binder (*K*_d_ ~10 μM). Briefly, N-terminal extensions of 11 to 16 amino acids were fused to linkers (GSEAK, GSEK, or GSK) and appended to the ip4_mutG coding sequence. Rational designs and controls (flexible N-terminal extensions) were incorporated into the library, ordered as oligonucleotide pools (oPools, IDT), and cloned into the v4 series plasmid for transformation into SB39 cells. Library selection was performed in 2 experimental setups: (a) short batch selection and (b) prolonged continuous selection in a turbidostat (Chi.Bio [61]).

In the batch selection, induced cultures diluted to OD_600_ = 0.05 were exposed to kanamycin concentrations ranging from 0 to 348 μg/ml. After 4.5 to 5 h, only cultures exhibiting a decrease in OD_600_ of ≥0.5 relative to antibiotic-free controls were subjected to NGS analysis. In the turbidostat setup, induced cultures at OD_600_ = 0.05 were exposed to kanamycin (209 μg/ml; 120% MIC_50_ of ASF1B_N_–ip3 in the same vector). Cultures were maintained at OD_600_ 0.4 for 21 or 29 h, followed by an additional 3-h growth period after sample collection, totaling 24 and 32 h.

Plasmids extracted from samples taken before (*T*_0_) and after selection (*T*_F_) were sequenced by NGS (Illumina), and reads corresponding to expected sequences were quantified to assess relative frequencies and enrichment.

### Expression and purification of ASF1’s binders

DNA sequences encoding T32_Kan[209]_top1, Kan[348]_top1, and Kan[348]_top2 peptides were cloned into pSMT3 plasmid as a (His)_6_–SUMO–peptide fusion proteins and expressed in *E. coli* BL21(DE3) Star. Protein expression was induced by 0.5 mM IPTG at 37 °C for 3 h. Cells were collected by centrifugation and were then resuspended in the lysis buffer (50 mM tris-HCl [pH 8], 1% Triton X-100, 500 mM NaCl, and 5% glycerol) supplemented with proteinase inhibitors (pefabloc [100 μg/ml], pepstatine [1 μg/ml], leupeptine [0.5 μg/ml], chymostatine [1 μg/ml], 1 mM phenylmethylsulfonyl fluoride, and aprotinine [8 μg/ml]), 250 μM dithiothreitol and 1× Roche EDTA-free complete protease inhibitor cocktail. Bacterial lysis was performed using a French press on frozen pellets. Lysates were immediately treated with Benzonase (12 U/ml) and 1 mM MgCl_2_, incubated at 4 °C for 15 min, and then centrifuged at 20,000 rpm for 20 min. Affinity purification was performed using a nickel-charged nitrilotriacetic acid agarose column (MACHEREY-NAGEL). After elution (50 mM tris [pH 8], 150 mM NaCl, and 300 mM imidazole), purified SUMO protease was added at a 1:40 mass ratio (protease:peptide) and incubated at 30 °C for 1 h to cleave the fusion. Peptides were further purified on a Resource RPC 3 ml column (Cytiva) and eluted via acetonitrile gradient, then lyophilized overnight using a SpeedVac system, and resuspended in water. Final purification was achieved by size exclusion chromatography using a Superdex 30 10/300 GL column (GE Healthcare) in 50 mM tris-HCl (pH 7.5). Peptide mass was confirmed by liquid chromatography–mass spectrometry. Fractions containing intact peptide were pooled and stored at −20 °C.

### Expression and purification of ASF1

Recombinant human ASF1A_N_ (residues 1 to 156) was produced in *E. coli* as a (His)_6_–glutathione *S*-transferase (GST)–tobacco etch virus cleavage site–ASF1 fusion protein using the pETM30 plasmid. Soluble (His)_6_-tagged GST fusion protein was purified on reduced glutathione agarose beads (Sigma-Aldrich). After overnight cleavage at room temperature with recombinant (His)_6_–tobacco etch virus protease, the (His)_6_–GST tag and protease were captured on a nickel-charged nitrilotriacetic acid agarose column (MACHEREY-NAGEL). The flow-through fraction containing ASF1A_N_ protein was further purified by anion exchange chromatography using a Resource Q 6 ml column (GE Healthcare). The protein was concentrated in an Amicon device (Millipore), and the buffer was exchanged to 50 mM tris-HCl (pH 7.5). Unlabeled hASF1A_N_ for ITC experiments was purified from cells grown in LB medium, whereas uniformly ^15^N- and/or ^15^N–^13^C-labeled ASF1A_N_ was purified from bacteria grown in M9 minimal medium supplemented with (^15^NH_4_)Cl (0.5 g/l; Eurisotop) as the sole nitrogen source and ^13^C-glucose (2 g/l) as carbon source.

### Isothermal titration calorimetry

ITC experiments were performed on a VP-ITC titration calorimeter (Microcal/Malvern) at 20 °C using 50 mM tris-HCl buffer (pH 7.5). Concentrations of ASF1 and binders ranged from 5 to 90 μM. Optimal ASF1:binder ratios were chosen to achieve clear transition curves and minimize fitting errors. Both proteins were prepared in the same buffer and degassed for 5 min by sonication or vacuum. After equilibrating the sample cell at 293.15 K, 10 μl of aliquots of binder solution (total of 30 injections) were injected into the ASF1 solution at 180-s intervals with the syringe rotating at 310 rpm until saturation was reached. Raw data were processed using Origin 7.0 software (OriginLab, Malvern) applying the One Set of Sites binding model. Experiments were performed in duplicate, and representative curves for each binder are shown in Fig. S23.

### Chemical shift mapping of ASF1A_N_ upon peptide binding

The binding mode of binders was assessed using NMR spectroscopy by monitoring amide chemical shifts of ASF1A_N_ residues. NMR experiments were performed at 293 K on Bruker NEO 700-MHz spectrometer equipped with a cryoprobe (Bruker). Purified uniformly labeled ^15^N hASF1A_N_ (1 to 156) was diluted in the NMR buffer comprising 50 mM tris-HCl (pH 7.5), 0.1 mM EDTA, 0.1 mM dextran sulfate sodium (DSS), 0.1 mM NaN_3_, protease inhibitor cocktail (per the manufacturer’s instructions, Roche), and 10% D_2_O. Proton chemical shifts (in parts per million) were referenced relative to internal DSS, and ^15^N reference was set indirectly relative to DSS using frequency ratios [18]. NMR data were processed using Topspin (Bruker) and analyzed using Sparky (T. D. Goddard and D. G. Kneller, University of California San Francisco). Amide assignments were adopted from previous studies [62]. An excess of T32_Kan[209]_top1 binder was added at a ratio 1:2 and a 2-dimensional ^1^H–^15^N SOFAST HMQC (heteronuclear multiple-quantum coherence) spectrum was recorded In the formula, and the NMR spectra are represented in Fig. S24. Chemical shift perturbations were quantified for all resonances using ^13^C–^15^N-labeled hASF1A_N_ in presence of T32_Kan[209]_top1. Chemical shift variation was calculated with the following formula:

$$\Delta \delta =\sqrt{{\left(\delta_{\mathrm{HN}}^{b}-\delta_{\mathrm{HN}}^{f}\right)}^{2}+{\left(0.17\left(\delta_{N}^{b}-\delta_{N}^{f}\right)\right)}^{2}}$$ (4)

In the formula, *δ* represents the measured chemical shift value; *b* and *f* denote the bound or free forms, respectively, while HN or N to the amide proton or nitrogen atoms, respectively. The factor 0.17 is a scaling constant used to normalize proton and nitrogen chemical shift changes (expressed in parts per million). The calculated chemical shift variations were plotted as a function of residue number for all 156 residues of hASF1A_N_ (1 to 156) (Fig. 5).

### Statistical analyses

#### Engineering experiments

Flow cytometry and MIC_50_ results were obtained from 3 independent biological replicates (*n* = 3). The *R*^2^ was used to evaluate the correlation between *K*_d_ and MFI or MIC_50_. Comparisons of MFIs or MIC_50_ values at different *K*_d_ values were performed using a one-tailed Student’s *t* test assuming unequal variances (based on *F*-test results), with significance defined as *P* < 0.05. The CV was used as a proxy for stochasticity, defined as the ratio of the standard deviation to the mean of measurements, expressed as a percentage.

#### Application experiments

In the context of binders’ optimization experiment, Pearson correlation coefficients (*r*) were used to assess correlations of variant frequencies or enrichment under different selection pressures. Only variants present in all conditions were used (*n* = 734 of 1,134 variants) were included in these analyses.

## Data Availability

The atomic coordinates of the ASF1 complexes with ip3 and HIRA are available in the PDB under accession codes 6f0g and 2i32, respectively. Materials, source data, and software used in this study are provided. Key plasmids utilized herein have been deposited at Addgene under IDs 235097 to 235120. The engineered strain SB39 is also available through Addgene (ID: 235121). NGS data, additional results, Python code, and notebooks for data analysis and figure generation are accessible at Zenodo: https://zenodo.org/records/15690360. Interface mapping results were analyzed using the mave2imap pipeline, which is available on GitHub (https://github.com/synth-bio-evo/mave2imap) and Pypi (https://pypi.org/project/mave2imap/). Additional information about designs and data analyses is provided in the Supplementary Materials.
